# Supplementary material for: A Rapid Method for Sequencing Double-Stranded RNAs Purified from Yeasts and the Identification of a Potent K1 Killer Toxin Isolated from Saccharomyces cerevisiae
Source: Viruses. 2019 Jan 16;11(1):70. doi: 10.3390/v11010070 (PMC6356530; doi:10.3390/v11010070)
Supplement: Supplementary file 1 [file viruses-11-00070-s001.zip › 3-supplementary tables and figures.pdf]

viruses

Supplementary figures

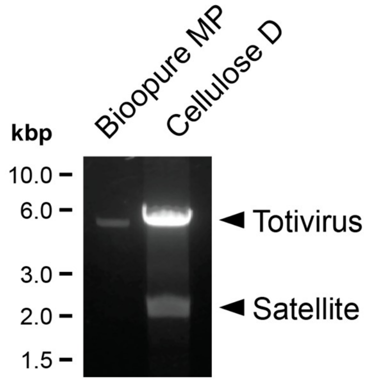

Figure 1. Comparing methods for dsRNA extraction from *Saccharomyces* yeasts. A 0.8% agarose gel comparing the extraction of dsRNAs from *S. cerevisiae* strain BJH001 using two different solvent extraction and chromatography methods. Lane 1, Bioopure MP (guanidinium thiocyanate and phenol) and silica spin column extraction method [1]. Lane 2, acid phenol and cellulose powder extraction method [2].

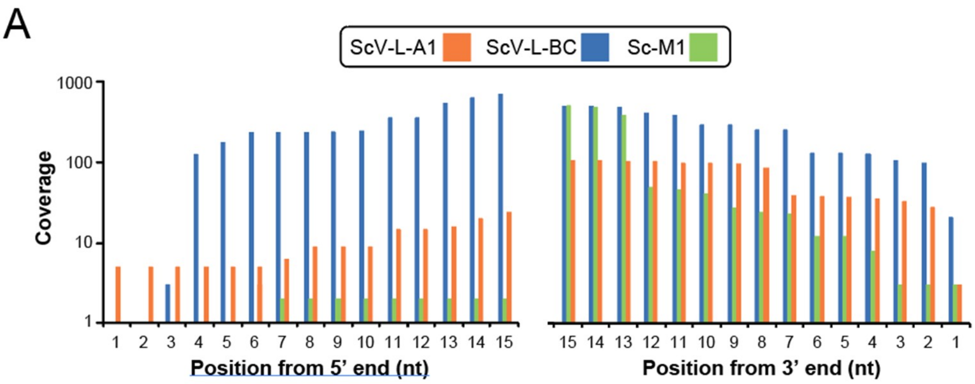

B

dsRNA	Total % GC	5' %GC	5' coverage	3' %GC	3' coverage
ScV-L-A1	46	7	10	30	67
ScV-L-BC	42	47	272	40	264
Sc-M1	45	27	1.2	33	107

Figure 2. Resolution of the 5′ and 3′ termini of dsRNAs using NGS. Log-scale coverage of sequence reads mapped onto the terminal 15 base pairs of ScV-L-A1, ScV-L-BC, and ScV-M1. The terminal 5′ two base pairs of ScV-L-BC were mapped by a single read. To resolve the A/U-rich 5′ terminus of ScV-L-A1 masking was turned off to enable read mapping. (b) Percentage GC content and average read coverage of the terminal 15 bp of dsRNAs isolated from *S. cerevisiae* BJH001.

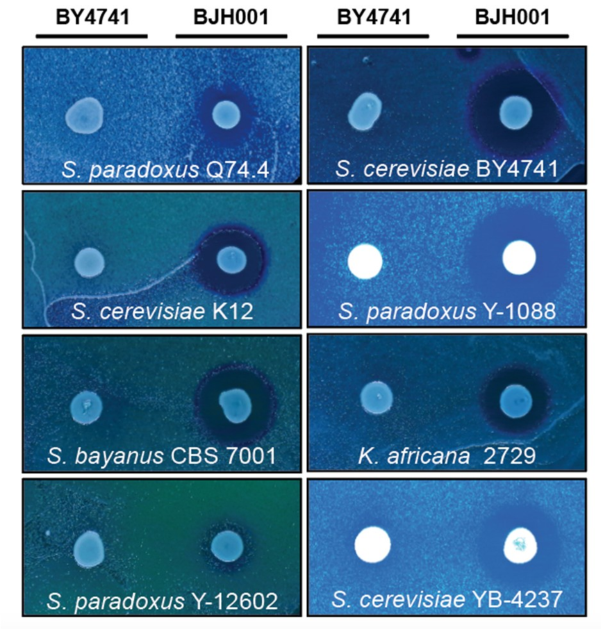

Figure 3. ***S. cerevisiae* BJH001 produces a killer toxin that can inhibit the growth of different yeast strains and species.** Killer toxin production on agar plates creates zones of growth inhibition around *S. cerevisiae* BJH001, but not the non-killer yeast *S. cerevisiae* BY4741.

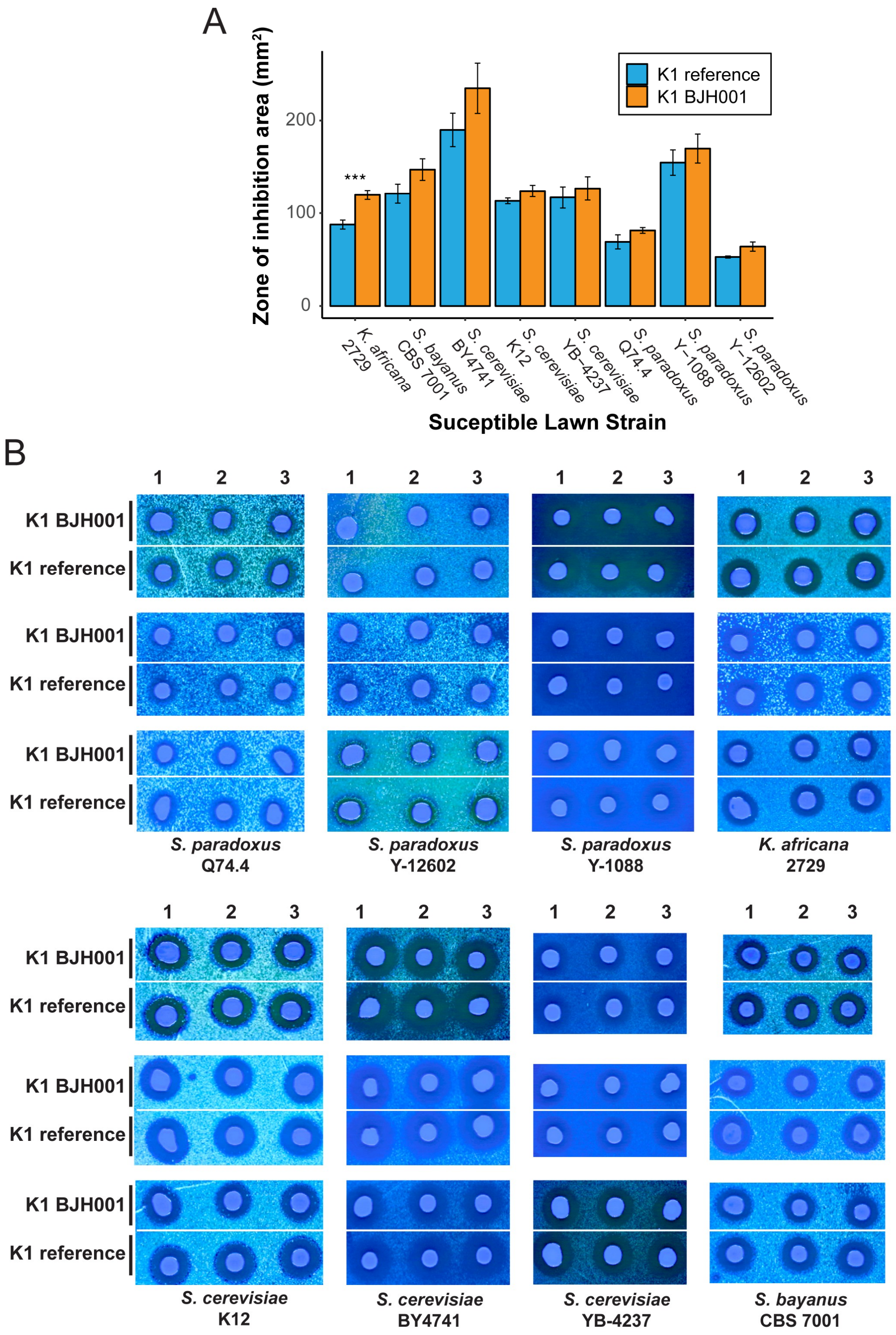

Figure 4. Mutations within the K1 gene increase the ability of the K1 killer toxin to inhibit the growth of *K. africana in vitro*. (a) The area of growth inhibition around K1-expressing *S. cerevisiae* challenged with different strains of K1-sensitive yeasts measured in mm^2. Asterisks are indicative of a significant difference in the mean zone of inhibition area (T-test, two-tailed, $p<0.01$). (b) Images of isogenic non-killer yeast strains expressing K1 killer toxins derived from the K1 reference sequence and K1 derived from *S. cerevisiae* BJH001 on agar seeded with yeasts known to be sensitive to K1 killer toxins. Three technical replicates are shown per image, and three independent replicates for each K1-sensitive yeast.

Supplementary Tables

Table 1. Primers used in this study.

Primer Name	Amplification Target	Primer Sequence (5′ to 3′)
Anchored Oligo(dT)$_{20}$	Poly(A) tracts	NV(dT)$_{20}$
PRX540	ScV-L-A1-specific primer	AAGATATTCGGAGTTGGTGATGACG
PRX541	ScV-L-A1-specific primer	TCTCCGAAATTTTTCCAGACTTTATAAGC
PRY691	ScV-L-BC-specific primers	CTCAAACGTTGGTCTCACCTGTACC
PRY692	ScV-L-BC-specific primers	CACTGAATGGTAAAACAGCATGTCG
PRX536	ScV-M1-specific primer	GCGATGCAGGTGTAGTAATCTTTGG
PRX537	ScV-M1-specific primer	AGTAGAAATGTCACGACGAGCAACG
PRUI1	ScV-M1-specific primer	GAGTTATCGCATCAGAGGTCAGACAC
PRUI2	ScV-M1-specific primer	GATGCCCTAGTGGCCTGTGTC
PRUI132	ScV-L-A1-specific primer	GTAAACGTAATCGAACCCTCACACG
PRUI133	ScV-L-A1-specific primer	ACCGACCCATATTGCTCTAGAATCC

Table 2. Yeast strains and species used in this study.

Species	Strain	Genotype [viruses]	Source
S. cerevisiae	BJH001	MATa, *his3Δ200 leu2Δ0 met15Δ0 trp1Δ63 ura3Δ0* [ScV-L-A1+, ScV-M1+, ScV-L-BC]	[1]
S. cerevisiae	BY4741	MATa, *his3Δ1 leu2Δ0 met15Δ0 ura3Δ0*	[3]
S. paradoxus	Q74.4	Matα, *ho::HygMX, ura3::KanMX-Barcode*	[4]
S. cerevisiae	Y8.5	Matα, *ho::HygMX, ura3::KanMX-Barcode*	[4]
S. cerevisiae	Montrachet	-	Red Star Yeast Company, LLC
S. cerevisiae	EC-1118	-	Lallemand Inc.
S. cerevisiae	BE-256	-	Fermentis (Lesaffre Group)
K. africana	2729	-	National collection of yeast cultures (NCYC)
S. cerevisiae	K12	-	Dr. Justin Fay
S. paradoxus	CBS12357	-	CBS-KNAW culture collection
S. bayanus	CBS7001	-	CBS-KNAW culture collection
S. cerevisiae	YB-4237	-	ARS culture collection (NRRL)
S. paradoxus	Y-12602	-	ARS culture collection (NRRL)
S. paradoxus	Y-1088	-	ARS culture collection (NRRL)

Supplementary files

File S1. The DNA sequences of the plasmids used in this study.
File S2. Mutations identified within dsRNAs extracted and sequenced in this study.
File S3. Raw images of agarose gels presented in this study.

References

1. Rowley, P.A.; Ho, B.; Bushong, S.; Johnson, A.; Sawyer, S.L. *XRN1* Is a Species-Specific Virus Restriction Factor in Yeasts. *PLoS Pathog.* **2016**, *12*, e1005890.
2. Okada, R.; Kiyota, E.; Moriyama, H.; Fukuhara, T.; Natsuaki, T. A simple and rapid method to purify viral dsRNA from plant and fungal tissue. *J. Gen. Plant Pathol.* **2015**, *81*, 103–107.
3. Brachmann, C.B.; Davies, A.; Cost, G.J.; Caputo, E.; Li, J.C.; Hieter, P.; Boeke, J.D. Designer deletion strains derived from *Saccharomyces cerevisiae* S288C: A useful set of strains and plasmids for PCR-mediated gene disruption and other applications. *Yeast* **1998**, *14*, 115–132.
4. Cubillos, F.A.; Louis, E.J.; Liti, G. Generation of a large set of genetically tractable haploid and diploid *Saccharomyces* strains. *FEMS Yeast Res.* **2009**, *9*, 1217–1225.
