# Supplementary material for: A Rapid Method for Sequencing Double-Stranded RNAs Purified from Yeasts and the Identification of a Potent K1 Killer Toxin Isolated from Saccharomyces cerevisiae
Source: Viruses. 2019 Jan 16;11(1):70. doi: 10.3390/v11010070 (PMC6356530; doi:10.3390/v11010070)
Supplement: Supplementary file 1 [file viruses-11-00070-s001.zip › File S3 - Raw gel images.pdf]

Supplementary file 3

Raw gel images:

Figure 1. The extraction of dsRNAs from dried and actively growing cultures of killer and non-killer Saccharomyces yeasts.

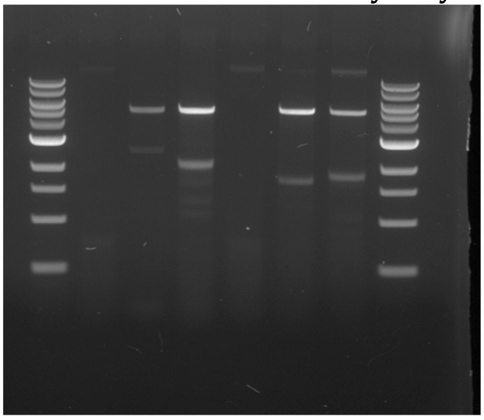

Lane 1 - Molecular weight marker (1kb ladder – New England Biolabs)
Lane 2 – CBS12357
Lane 3 – Y8.5
Lane 4 – BJH001
Lane 5 – BE-256
Lane 6 – EC-1118
Lane 7 – Montrachet
Lane 8 - Molecular weight marker (1kb ladder – New England Biolabs)

Figure 3B (inset). Next-generation sequencing of totiviruses and dsRNA satellites from S. cerevisiae strain BJH001.

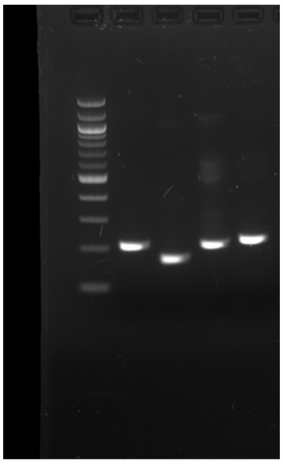

Lane 1 - Molecular weight marker (1kb ladder – New England Biolabs)
Lane 2 – RT-PCR amplification of ScV-M1

Lane 3 – RT-PCR amplification of ScV-L-BC
Lane 4 – RT-PCR amplification of ScV-L-BC
Lane 5 – RT-PCR amplification of ScV-L-A1

Figure S1. Comparing methods for dsRNA extraction from *Saccharomyces* yeasts.

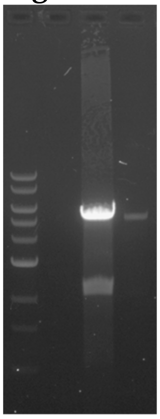

Lane 1 - Molecular weight marker (1kb ladder – New England Biolabs)
Lane 2 – No sample
Lane 3 - Bioopure MP and silica spin column extraction method
Lane 4 - Acid phenol and cellulose powder extraction method
